# Determination of Bioelectrical Impedance Thresholds for Early Detection of Breast Cancer-related Lymphedema

**DOI:** 10.7150/ijms.53812

**Published:** 2021-06-11

**Authors:** Siyao Liu, Quanping Zhao, Xinmei Ren, Ying Cui, Houpu Yang, Siyuan Wang, Miao Liu, Shu Wang

**Affiliations:** Breast Center, Peking University People's Hospital, Beijing, China.

**Keywords:** lymphedema, breast cancer, bioelectrical impedance

## Abstract

**Background:** Bioelectrical impedance technology is a common technique used for the early detection of breast cancer-related lymphedema (BCRL). However, studies on the threshold value established by Inbody 720 device (Biospace, Korea) have been extremely limited. We aimed to determine its reference range and cutoff values.

**Methods:** All patients were recruited from October 2017 to October 2019 at the Peking University People's Hospital Breast Center. In total, 82 patients with unilateral BCRL and 1305 healthy subjects were recruited in this study. We measured the extracellular fluid (ECF) ratio, extracellular water (ECW) ratio, as well as the single-frequency bioimpedance analysis (SFBIA) ratios at 1 and 5 kHz with the Inbody 720 device. The Youden index-based cutoff points, mean + 2SD and mean + 3SD values of these four indicators for both dominant and nondominant arms were also calculated.

**Results:** Data were collected from 1387 women, including healthy subjects and patients with lymphedema. All statistical analyses were performed with SPSS. Significant differences were found between the two groups in the ECW, ECF, and SFBIA ratios. For the dominant affected arms, the Youden index-based cutoff points for the ECF, ECW, as well as SFBIA ratios at 1 and 5 kHz were 1.009, 1.008, 1.068, and 1.068, respectively. For the nondominant affected arms, the Youden index-based cutoff points were 1.014, 1.013, 1.047, and 1.048, respectively. The mean + 2 standard deviations (SD) and mean + 3SD values were also calculated.

**Conclusions:** We determined the Youden index-based cutoff points, mean + 2SD and mean + 3SD values of the ECF, ECW, as well as SFBIA ratios at 1 and 5 kHz for both dominant and nondominant arms with data from 1305 healthy subjects. Next, the Youden index-based cutoff points, the mean + 2SD and mean + 3SD values were used to recognize patients with lymphedema. We found that the Youden index-based cutoff points and the mean + 2SD showed similar identification capacity on lymphedema, and they seemed to distinguish more patients with lymphedema than mean + 3SD values.

## Introduction

Breast cancer-related lymphedema (BCRL) is a major long-term complication following breast cancer treatment, such as axillary lymph node dissection, taxane-based chemotherapy, and radiation 1. Studies have also demonstrated some nontreatment-related risk indicators for BCRL, including the body mass index (BMI) at the time of diagnosis, subclinical lymphedema, and cellulitis on the side of treatment 1, 2. It is a chronic debilitating disease that manifested by an abnormal accumulation of protein-rich fluid in tissues that reflects the “relative” imbalance between the lymphatic transport capacity and lymphatic load 3. A limited number of investigations have also suggested that genetic susceptibility may play a role in the pathogenesis of lymphedema following breast cancer therapy 4-6.

The diagnosis of lymphedema is challenging, especially in the early stages (0 or 1) of the disease, with diverse definitions and objective instruments available for diagnostic assessment 3, 7, 8. Early diagnosis and intervention are important because the prognosis of lymphedema is poor and more complications will appear as the disease progresses. However, early-stage diagnosis presupposes the suitable tools capable of identifying individuals with high risk of lymphedema (LE) at the earliest opportunity 9. The ideal measurement tool should be easy to use, hygienic, noninvasive, reliable, cost effective, quantifiable and reproducible 3. Conventionally, lymphedema has been defined as increases in limb circumference by a 2 cm, increases in limb volume by 200 mL, or limb volume changes of 10% pertaining to the comparison of affected (or lymphedematous) and unaffected limbs 10. However, these indirect assessment methods cannot distinguish bone, fat, muscle, or other soft tissues from extracellular or lymph fluid.

The advent of bioimpedance measurements has offset their shortcomings. Compared with circumferential measurements 11, volume displacement 12, 13, perometer 14 13, lymphography 2, 15-17 and lymphoscintigraphy 18, a variety of publications have demonstrated the ability of bioimpedance to diagnose lymphedema. Bioimpedance is a measure of impedance to the electric current which flows through the human body or a body part 19, and operates based on the principle that the impedance at zero frequency is a direct representative of extracellular water (ECW) volume 20. Because of excessive accumulation of lymph fluid, lymphedema usually results in an overall increase in the total amount of ECW in the affected limb. As the volume of ECW increases, the impedance to the current decreases 10. In addition to ECW, the concept of extracellular fluid (ECF) has also appeared in previous studies because they have similar meanings and are easily confused. In fact, they are different expressions of body composition analysis at different levels 21. ECF is not only related to ECW, but also related to solids. According to the earliest research, the impedance to current flow measured at zero frequency is the measure of extracellular water volume 22. Therefore, with this theory, ECW is now considered as a better indicator.

Based on the principle of bioimpedance, although it is best to measure the impedance at zero frequency, practical limitations prevent a direct measure of impedance at zero frequency. There are two possible measurement techniques that can provide the best estimate of the impedance at zero frequency. One is the bioimpedance spectroscopy (BIS) device, which measures impedance over a range of frequencies and extrapolates the data to zero frequency through the Cole modeling method. The alternative and less expensive method, bioimpedance analysis (BIA) device, measures impedance at a single constant frequency in the low frequency range. The assumption is that these low frequencies are close to zero that the values for impedance at these frequencies will be similar to those extrapolated at zero frequency 23. Impedance values at 1 kHz and 5kHz are commonly used, which were determined by the principle of machine. The former one is commonly used and is the earliest bioimpedance device used to detect lymphedema in 1992 22, 24. The latter one represented by Inbody 720 was extensively used to monitor the therapeutic effect of lymphedema 11, 25, 26. However, there are few studies on the diagnosis and diagnostic threshold of lymphedema. Thus, it is particularly important to develop a reference impedance ratio for Inbody 720. In 2018, Jung et al. first reported the diagnostic criteria of Inbody 720 27. However, our study is based on a larger sample to determine the cutoff values for the diagnosis of lymphedema and to verify the ability of these values in detecting lymphedema.

## Methods

### Participants

This study was a prospective clinical trial conducted at Peking University People's Hospital (PKUPH) and was registered on ClinicalTrials.gov. Its clinical trial registration number was NCT02691624. Ethical permission to collect these data was obtained from the PKUPH.

Preoperative subjects diagnosed with breast cancer at the Breast Center of PKUPH who completed preoperative bioimpedance measurement between October 2017 and October 2019 were enrolled as relatively healthy controls in this study. In order to obtain data on relatively healthy subjects, we adopted strict inclusion and exclusion criteria. We strictly excluded all possible causes of lymphedema. Subjects whose clinical stage of lymph node is N2 or higher were excluded. Subjects with lymphedema-related diseases or kidney disease, inflammatory breast cancer, a history of axillary surgery, radiotherapy to the upper limbs or the chest wall, soft tissue infection, pregnancy, congestive heart-failure, administration of diuretics (which may have significantly changed the hydration status) and implanted devices (e.g., pacemakers), were also excluded. 1305 participants were recruited as the healthy controls.

We recruited breast cancer patients with a diagnosis of unilateral BCRL from the PKUPH between October 2017 and October 2019. All participants had undergone breast and axillary surgeries. BCRL was defined as increases in ipsilateral arm measurements of 2 cm or greater compared with contralateral arm measurements at locations at any site 3, 28. A total of 82 patients with BCRL were recruited. Patients with a history of bilateral axillary surgeries were excluded from the study. We collected data on age (in years), dominant limb, BMI (kg/m^2^), time (year, month, day), and type of surgery (mastectomy or lumpectomy).

### Bioimpedance measurements

All patients underwent bioimpedance measurements (Inbody 720, South Korea) to acquire the calculated edema ratio 1 (extracellular water (ECW)/total cellular water (TCW)), the calculated edema ratio 2 (extracellular fluid (ECF)/total cellular fluid (TCF)), and the impedence values of SFBIA at 1 and 5 kHz for both upper extremities. Measurements were performed according to the manufacturer's instructions. The participant stood on two metal plates, with a current-driven electrode under the ball of each foot, and a voltage-sensing electrode under the heel of each foot. When fingers were bent to hold the handle, the hand electrodes were the current-driven electrodes under the ball of the thumb with the voltage-sensing electrode on the palm 29.

### Evaluation criteria

The edema ratio 1 or edema ratio 2 was used to calculate the ECW or ECF ratio. The impedence value was used to calculate the SFBIA ratio. For patients, the calculated ECF (edema ratio 2_affected side_/ edema ratio 2_unaffected side_) or ECW (edema ratio 1_affected side_/ edema ratio 1_unaffected side_) ratio is defined as a ratio of the affected to the unaffected side, and the SFBIA ratio is defined as a ratio of the unaffected to the affected side 9. For healthy women, arm dominance was also considered. The calculated ECF (edema ratio 2_dominant side_/edema ratio 2_nondominant side_) or ECW (edema ratio 1_dominant side_/edema ratio 1_nondominant side_) ratio of dominant arm was defined as a ratio of the dominant side to the nondominant side, and the SFBIA ratio was defined as a ratio of the nondominant to the dominant side. The data of nondominant arm was opposite. Apply data from healthy women to the patient group to verify whether these values could be used to detect lymphedema. If the dominant arm was affected in the patient group, then the data should be compared to the dominant data in healthy women.

### Statistical analyses

All statistical analyses were performed with SPSS (version 24, IBM Corporation, Armonk, NY, USA). We used the Student's t-test to compare the mean values of BMI, age, calculated ECF, ECW, and SFBIA ratios between the patient and the control groups, and also compared the impedance values at 1 kHz and 5 kHz between the dominant arms and the nondominant arms in healthy subjects. Additionally, the chi-square test was used to analyze the arm dominance between the patient group and the control group. The most commonly used optimality criterion for cutoff point selection in the context of ROC curve analysis is the maximum of the Youden index, which is named as the Youden index-based cutoff points in our study. The Youden index-based cutoff points, mean + 2SD and mean + 3SD values for the calculated ECF ratio, ECW ratio, as well as SFBIA ratios at 1 kHz and 5 kHz were calculated. Sensitivity and specificity of the Youden index-based cutoff points, mean + 2SD and mean + 3SD values were also calculated. P < 0.05 was considered statistically significant.

## Results

### Patients

In total, 1387 women were recruited in our study from October 2017 to October 2019. There were 1305 participants in the control group and 82 participants in the lymphedema group. Participant demographic characteristics are presented in Table 1. The majority (92.9%) of participants were right-handed. There were statistically significant differences between the two groups in terms of age and BMI, but there was no statistically significant difference in the dominant arm. Women in the patient group had significantly higher BMI and age compared with the control group.

### Limb impedance measurements

We calculated the ECF ratios, ECW ratios, as well as SFBIA ratios at 1 and 5 kHz. Impedance values of healthy participants' stratified according to limb dominance are presented in Table 2. Results showed a statistically significant difference between the two groups (P < 0.001). There were significant differences in ECF ratios, ECW ratios, as well as SFBIA ratios at 1 and 5 kHz between patients with lymphedema and healthy controls (Table 3).

### Cutoff values

To distinguish patients with lymphedema from healthy women, we calculated the mean +2SD and mean + 3SD values (Table 4) of healthy women, and analyzed the ROC curve for lymphedema diagnosis (Table 5). The Youden index-based cutoff points, mean + 2SD, and mean + 3SD values derived from the ECF ratio for the dominant affected arms were 1.009, 1.009, and 1.014, respectively. Furthermore, for the nondominant affected arms, these three values were 1.014, 1.012, and 1.018, respectively. The Youden index-based cutoff points, mean + 2SD, and mean + 3SD values derived from the ECW ratio for the dominant affected arms were 1.008, 1.008, and 1.013, respectively. Furthermore, for the nondominant affected arms, these three values were 1.013, 1.012, and 1.016, respectively. The Youden index-based cutoff points, mean + 2SD, and mean + 3SD values derived from the SFBIA ratio at 1 kHz for the dominant affected arms were 1.068, 1.067, and 1.095, respectively. In addition, for the nondominant affected arms, these three values were 1.047, 1.043, and 1.070, respectively. The Youden index-based cutoff points, mean + 2SD, and mean + 3SD values derived from the SFBIA ratio at 5 kHz for the dominant affected arms were 1.068, 1.068, and 1.096, respectively. Moreover, for the nondominant affected arms, these three values were 1.048, 1.044, and 1.071, respectively. The sensitivity and specificity values are listed in Table 4, Table 5.

In addition, the values of the area under the curve (AUC) are presented in Table 5. When the Youden index-based cutoff points, mean + 2SD, and mean + 3SD values are applied to identify patients with lymphedema, we found that the Youden index-based cutoff points and the mean + 2SD showed similar identification capacity on lymphedema, and they seemed to distinguish more patients with lymphedema than mean + 3SD values (Table 6).

## Discussion

In 1992, Ward et al. demonstrated that the mean impedances were significantly different between those of the lymphedema and control groups 24. In 2001, Cornish et al. published a study, which involved 162 participants, that determined the threshold variation for the early detection of lymphedema. This threshold was set as 3SD from the baseline measures 30. More recently, 2SD threshold is being recommended because it can provide better sensitivity 22.

Nowadays, Inbody 720 was also a commonly used impedance device for lymphedema research. However, only a few studies were conducted on the threshold criteria for the detection of lymphedema. Jung et al. 27 determined the cutoff, mean + 2SD and mean + 3SD values of the ECF volume, SFBIA at 1 and 5 kHz frequencies for both dominant and nondominant arms using data from 70 patients with unilateral BCRL and 643 healthy subjects in South Korea.

Our study has also obtained data on the maximum sample size of the calculated edema ratio 1 (ECW/TCW), the calculated edema ratio 2 (ECF/TCF), as well as SFBIA at 1 and 5 kHz for the assessment of BCRL using Inbody 720. The criteria ratios in our study were similar to the results of Jung et al. 26. The difference is that we added the reference value of the ECW ratio. In principle, we think that ECW ratio may be a better indicator. Because ECW ratio provides information about the amount of water in the extracellular environment and ECF ratio is related to added protein and minerals 11. They are actually different expressions of different levels of body composition analysis 21. In our research, we have provided reference values for ECW and ECF, but in clinical practice, which of these two indicators is better requires more research and exploration.

To evaluate the practical ability of different threshold criteria in detecting lymphedema in this study, existing data associated with the patients group were classified according to these three sets of criteria with four evaluation indicators. Overall, based on the results of this study, the ability of the Youden index-based cutoff point is relatively similar with mean + 2SD in detecting lymphedema. Taking the Youden index-based cutoff point as an example, if the dominant hand is affected, its sensitivity and specificity for diagnosing lymphedema are 88.9% and 98.1%, respectively. According to previous research, BIS is used for the diagnosis of lymphedema due to its high specificity (80-99%). However, due to the wide range of sensitivities observed (30-100%), its sensitivity is still controversial 15. The wide range of sensitivity and specificity found in different studies may be due to different BIS thresholds and different comparison measurements selected in the literature 22. Lim et al. 31 determined the cutoff values of SFBIA ratio at 1 and 5 kHz are 1.049 and 1.047, respectively. In their study, the SFBIA ratio at 5 kHz showed better performance compared to 1 kHz, with a specificity of 95.15% and a sensitivity of 63.64%. As differences in the amounts of fluid in the dominant and non-dominant limbs have been found, it has been recognized that different thresholds are needed whether the at-risk limb is dominant or non-dominant 27, 32, However, this was not considered in the Lim study, possibly explaining the difference in findings from the current study. In addition, in our study, the impedance of the dominant arm was significantly lower (P < 0.001) compared with the nondominant arm that reflected the necessity of distinguishing a dominant arm during the development of reference standards.

As far as we know, our research is a study on the largest sample size of the cutoff value of Inbody 720. Compared with the study of Jung et al. 26, our research showed that the SFBIA ratios of healthy controls as well as ECF ratios of patients with lymphedema were similar to their results, but the SFBIA ratios of patients with lymphedema in our study is larger. Our research results add an Inbody threshold, which can be used in context with other assessment and clinical findings when diagnosing lymphedema.

However, there are some limitations associated with our study. First, the number of patients with lymphedema was small compared with the control group. But the sample size of a similar study 27 on bioimpedance measurements was smaller than that of our study. Second, the control population in our study is a relatively healthy population, not purely healthy volunteers. This large sample of subjects comes from our preoperative breast cancer patients. Although we have adopted strict inclusion and exclusion criteria to ensure that they are relatively healthy population compared with lymphedema patients, this is still a main limitation of our study. Third, our device could not directly measure the ECF volume, but could only calculate the ratio of ECF to the TCF. If we could directly measure ECF volume, more accurate results could be expected. Fourth, the staging of patients with lymphedema in our study may be slightly later than in Jung's study, which leads to a larger value of SFBIA ratio in the patient group in table 3. Fifth, according to the classification of the ISL for lymphedema 33, our study included three patients with stage III that encompassed trophic skin changes, such as the alterations in skin character and thickness, further deposition of fat and the presence of fibrosis may not be detectable by bioimpedance measurements that may lead to less accurate results. Finally, another limitation is the general challenge faced by lymphedema research, because we do not have an agreed reference standard for the detection of lymphedema. In addition to arm circumference measurement as the diagnostic criteria for lymphedema, there may be other diagnostic methods, such as lymphoscintigraphy and indocyanine green lymphography, which makes ROC/AUC as well as sensitivity and specificity analysis more challenging.

## Conclusions

In summary, new threshold criteria have been provided for the assessment of BCRL by Inbody 720. We determined the Youden index-based cutoff point, mean + 2SD, and mean + 3SD values of the ECF ratio, ECW ratio, as well as SFBIA ratios at 1 kHz and 5 kHz for both dominant and nondominant arms using data from 1387 subjects. When the Youden index-based cutoff points, mean + 2SD, and mean + 3SD values are applied to identify patients with lymphedema, we found that the Youden index-based cutoff points and the mean + 2SD showed similar identification capacity on lymphedema, and they seemed to distinguish more patients with lymphedema than mean + 3SD values.

## Figures and Tables

**Table 1:** Demographic characteristics of participants

Variable	Patient group (n = 82)	Control group (n = 1305)	95% CI of the difference	t score	P value
Age	61.4 ± 10.8	53.7 ± 12.8	5.264-10.208	6.211	0.000
BMI	25.3 ± 3.3	24.2 ± 3.5	0.375-1.927	2.911	0.004
Dominant Arm			-	-	0.154
Left	9	89	-	-	-
Right	73	1216	-	-	-
CM difference (cm)	3.9+2.0	-	-	-	-
LE duration (month)	17.1+31.0	-	-	-	-

BMI, body mass index; CI, confidence interval; LE: lymphedema; CM, circumference.

**Table 2:** Impedance values of healthy participants

	Nondominant arm	Dominant arm	95% CI of the difference	t score	P value
**SFBIA values**					
1 kHz	404.9 ± 49.5	400.1 ± 48.2	4.169-5.351	15.807	0.000
5 kHz	395.7 ± 49.1	391.0 ± 47.8	4.065-5.234	15.618	0.000

SFBIA, single-frequency bioimpedance analysis; CI, confidence interval.

**Table 3:** Impedance ratios of patients with lymphedema and healthy controls

	Patient group (n = 82)	Control group (n = 1305)	95% CI of the difference	t score	P value
**ECF ratio**					
Dominant affected arm	1.032 ± 0.031	0.998 ± 0.005	0.025-0.044	7.301	0.000
Nondominant affected arm	1.041 ± 0.029	1.002 ± 0.005	0.029-0.049	8.137	0.000
**ECW ratio**					
Dominant affected arm	1.030 ± 0.029	0.998 ± 0.005	0.023-0.040	7.279	0.000
Nondominant affected arm	1.037 ± 0.027	1.002 ± 0.005	0.027-0.045	8.019	0.000
**SFBIA ratio at 1 kHz**					
Dominant affected arm	1.327 ± 0.362	1.012 ± 0.028	0.206-0.424	5.833	0.000
Nondominant affected arm	1.352 ± 0.239	0.989 ± 0.027	0.283-0.443	9.226	0.000
**SFBIA ratio at 5 kHz**					
Dominant affected arm	1.327 ± 0.362	1.012 ± 0.028	0.206-0.423	5.833	0.000
Nondominant affected arm	1.352 ± 0.239	0.989 ± 0.027	0.283-0.443	9.226	0.000

ECF, extracellular fluid; ECW, extracellular water; SFBIA, single-frequency bioimpedance analysis; CI, confidence interval.

**Table 4:** Mean + 2 standard deviation (SD) and mean + 3SD values used for the diagnosis of lymphedema

	Mean + 2SD	Sensitivity	Specificity	Mean + 3SD	Sensitivity	Specificity
**ECF ratio**						
Dominant affected arm	1.009	0.844	0.958	1.014	0.733	0.994
Nondominant affected arm	1.012	0.892	0.985	1.018	0.838	0.995
**ECW ratio**						
Dominant affected arm	1.008	0.8	0.981	1.013	0.711	0.996
Nondominant affected arm	1.012	0.892	0.983	1.016	0.811	0.996
**SFBIA ratio at 1 kHz**						
Dominant affected arm	1.067	0.889	0.984	1.095	0.778	1
Nondominant affected arm	1.043	0.892	0.973	1.070	0.865	0.995
**SFBIA ratio at 5 kHz**						
Dominant affected arm	1.068	0.889	0.98	1.096	0.756	0.999
Nondominant affected arm	1.044	0.892	0.971	1.071	0.865	0.996

SD, standard deviation; ECF, extracellular fluid; ECW, extracellular water; SFBIA, single frequency bioimpedance analysis.

**Table 5:** The Youden index-based cutoff points for the diagnosis of lymphedema

	AUC	95% CI	Youden cutoff	Sensitivity	Specificity
**ECF ratio**					
Dominant affected arm	0.939	0.888-0.990	1.009	0.844	0.959
Nondominant affected arm	0.925	0.846-1.000	1.014	0.892	0.986
**ECW ratio**					
Dominant affected arm	0.938	0.887-0.989	1.008	0.844	0.962
Nondominant affected arm	0.921	0.840-1.000	1.013	0.892	0.984
**SFBIA ratio at 1 kHz**					
Dominant affected arm	0.965	0.920-1.000	1.068	0.889	0.985
Nondominant affected arm	0.951	0.888-1.000	1.047	0.892	0.977
**SFBIA ratio at 5 kHz**					
Dominant affected arm	0.964	0.920-1.000	1.068	0.889	0.981
Nondominant affected arm	0.951	0.889-1.000	1.048	0.892	0.978

ECF, extracellular fluid; ECW, extracellular water; SFBIA, single-frequency bioimpedance analysis; AUC: area under curve; CI, confidence interval.

**Table 6:** Use of the Youden index-based cutoff points, mean + 2SD and mean + 3SD values to predict patients with lymphedema

	Patients ≥ Youden cutoff	Patients ≥ mean + 2SD	Patients ≥ mean + 3SD
**ECF ratio**			
Dominant affected arm	38/45 (84.4%)	37/45 (82.2%)	34/45 (75.6%)
Nondominant affected arm	33/37 (89.2%)	33/37 (89.2%)	31/37 (83.8%)
**ECW ratio**			
Dominant affected arm	38/45 (84.4%)	36/45 (80.0%)	33/45 (73.3%)
Nondominant affected arm	33/37 (89.2%)	33/37 (89.2%)	31/37 (83.8%)
**SFBIA ratio at 1 kHz**			
Dominant affected arm	40/45 (88.9%)	40/45 (88.9%)	34/45 (75.6%)
Nondominant affected arm	33/37 (89.2%)	33/37 (89.2%)	32/37 (83.8%)
**SFBIA ratio at 5 kHz**			
Dominant affected arm	40/45 (88.9%)	40/45 (88.9%)	34/45 (73.3%)
Nondominant affected arm	33/37 (89.2%)	33/37 (89.2%)	32/37 (83.8%)

SD, standard deviation; ECF, extracellular fluid; ECW, extracellular water; SFBIA, single frequency bioimpedance analysis.

## Abbreviations

ECF: extracellular fluid
ECW: extracellular water
TCW: total cellular water
TCF: total cellular fluid
SFBIA: single-frequency bioimpedance analysis
LE: lymphedema
BCRL: breast cancer-related lymphedema
SD: standard deviation
BIA: Bioelectrical impedance analysis
BIS: bioimpedance spectroscopy
PKUPH: Peking University People's Hospital
BMI: body mass index
ROC: receiver-operating characteristic curve
ISL: International Society of Lymphology
AUC: Area under curve
CI: confidence interval
CM: circumference

## References

[1] Gillespie TC, Sayegh HE, Brunelle CL (2018). Breast cancer-related lymphedema: risk factors, precautionary measures, and treatments. Gland Surg.

[2] Kayiran O, De La Cruz C, Tane K, Soran A (2017). Lymphedema: From diagnosis to treatment. Turkish Journal of Surgery.

[3] McLaughlin SA, Staley AC, Vicini F (2017). Considerations for Clinicians in the Diagnosis, Prevention, and Treatment of Breast Cancer-Related Lymphedema: Recommendations from a Multidisciplinary Expert ASBrS Panel: Part 1: Definitions, Assessments, Education, and Future Directions. Ann Surg Oncol.

[4] Azhar SH, Lim HY, Tan BK, Angeli V (2020). The Unresolved Pathophysiology of Lymphedema. Front Physiol.

[5] Suami H (2020). Anatomical Theories of the Pathophysiology of Cancer-Related Lymphoedema. Cancers (Basel).

[6] Visser J, van Geel M, Cornelissen AJM (2019). Breast Cancer-Related Lymphedema and Genetic Predisposition: A Systematic Review of the Literature. Lymphat Res Biol.

[7] O'Donnell TF Jr, Rasmussen JC, Sevick-Muraca EM (2017). New diagnostic modalities in the evaluation of lymphedema. J Vasc Surg Venous Lymphat Disord.

[8] Wiser I, Mehrara BJ, Coriddi M (2020). Preoperative Assessment of Upper Extremity Secondary Lymphedema. Cancers (Basel).

[9] Ward LC (2006). Bioelectrical impedance analysis: proven utility in lymphedema risk assessment and therapeutic monitoring. Lymphat Res Biol.

[10] Fu MR, Cleland CM, Guth AA (2013). L-dex ratio in detecting breast cancer-related lymphedema: reliability, sensitivity, and specificity. Lymphology.

[11] Kim L, Jeon JY, Sung IY (2011). Prediction of treatment outcome with bioimpedance measurements in breast cancer related lymphedema patients. Ann Rehabil Med.

[12] Barrio AV, Eaton A, Frazier TG (2015). A Prospective Validation Study of Bioimpedance with Volume Displacement in Early-Stage Breast Cancer Patients at Risk for Lymphedema. Ann Surg Oncol.

[13] Czerniec SA, Ward LC, Refshauge KM (2010). Assessment of breast cancer-related arm lymphedema-comparison of physical measurement methods and self-report. Cancer Invest.

[14] Ward LC, Czerniec S, Kilbreath SL (2009). Quantitative bioimpedance spectroscopy for the assessment of lymphoedema. Breast Cancer Res Treat.

[15] Qin ES, Bowen MJ, Chen WF (2018). Diagnostic accuracy of bioimpedance spectroscopy in patients with lymphedema: A retrospective cohort analysis. J Plast Reconstr Aesthet Surg.

[16] Executive Committee of the International Society of L (2020). The diagnosis and treatment of peripheral lymphedema: 2020 Consensus Document of the International Society of Lymphology. Lymphology.

[17] Rockson SG (2018). Diagnosis of Early and Subclinical Lymphedema Following Breast Cancer. Lymphat Res Biol.

[18] Coroneos CJ, Wong FC, DeSnyder SM (2019). Correlation of L-Dex Bioimpedance Spectroscopy with Limb Volume and Lymphatic Function in Lymphedema. Lymphat Res Biol.

[19] Hidding JT, Viehoff PB, Beurskens CH (2016). Measurement Properties of Instruments for Measuring of Lymphedema: Systematic Review. Phys Ther.

[20] Ward LC, Dylke E, Czerniec S (2011). Confirmation of the reference impedance ratios used for assessment of breast cancer-related lymphedema by bioelectrical impedance spectroscopy. Lymphat Res Biol.

[21] Wang ZM, Pierson RN Jr, Heymsfield SB (1992). The five-level model: a new approach to organizing body-composition research. Am J Clin Nutr.

[22] Dylke ES, Ward LC (2020). Three Decades of Bioelectrical Impedance Spectroscopy in Lymphedema Assessment: An Historical Perspective. Lymphatic Research and Biology.

[23] York SL, Ward LC, Czerniec S (2008). Single frequency versus bioimpedance spectroscopy for the assessment of lymphedema. Breast Cancer Research and Treatment.

[24] Ward LC, Bunce IH, Cornish BH (1992). Multi-frequency bioelectrical impedance augments the diagnosis and management of lymphoedema in post-mastectomy patients. Eur J Clin Invest.

[25] Carati CJ, Anderson SN, Gannon BJ, Piller NB (2003). Treatment of postmastectomy lymphedema with low-level laser therapy. Cancer.

[26] Riegerová J, Gába A, Valenta M, Chromý O (2010). Ratio Changes of Selected Components of Body Composition after Lymphatic Massage. Acta Universitatis Palackianae Olomucensis Gymnica.

[27] Jung M, Jeon JY, Yun GJ (2018). Reference values of bioelectrical impedance analysis for detecting breast cancer-related lymphedema. Medicine.

[28] Armer JM, Stewart BR (2010). Post-breast cancer lymphedema: incidence increases from 12 to 30 to 60 months. Lymphology.

[29] van Zanten M, Piller N, Ward LC (2016). Inter-Changeability of Impedance Devices for Lymphedema Assessment. Lymphatic Research and Biology.

[30] Cornish BH, Chapman M, Hirst C (2001). Early diagnosis of lymphedema using multiple frequency bioimpedance. Lymphology.

[31] Lim SM, Han Y, Kim SI, Park HS (2019). Utilization of bioelectrical impedance analysis for detection of lymphedema in breast Cancer survivors: a prospective cross sectional study. BMC Cancer.

[32] Wang H, Li D, Liuya J (2017). Reference Ranges Using Bioimpedance for Detection of Lymphedema in Chinese Women. Lymphatic Research and Biology.

[33] Executive C (2016). The Diagnosis and Treatment of Peripheral Lymphedema: 2016 Consensus Document of the International Society of Lymphology. Lymphology.

